# Local myalgia compared to myofascial pain with referral according to the DC/TMD: Axis I and II results

**DOI:** 10.1186/s12903-022-02048-x

**Published:** 2022-02-04

**Authors:** Orit Winocur-Arias, Pessia Friedman-Rubin, Kian Abu Ras, Larry Lockerman, Alona Emodi-Perlman, Tzvika Greenbaum, Shoshana Reiter

**Affiliations:** 1 Department of Oral Pathology, Oral Medicine, and Maxillofacial Imaging, The Maurice and Gabriela Goldschleger School of Dental Medicine, Sackler Faculty of Medicine, Tel Aviv University, Tel Aviv, Israel; 2 Department of Oral Rehabilitation, The Maurice and Gabriela Goldschleger School of Dental Medicine, Sackler Faculty of Medicine, Tel Aviv University, Tel Aviv, Israel; 3 The Maurice and Gabriela Goldschleger School of Dental Medicine, Tel Aviv University, Tel Aviv, Israel; 4 Department of Endodontics, The Maurice and Gabriela Goldschleger School of Dental Medicine, Sackler Faculty of Medicine, Tel Aviv University, Tel Aviv, Israel; 5 Department of Physical Therapy, Recanati School for Community Health Professions, Faculty of Health Sciences, Ben-Gurion University of the Negev, Beer Sheva, Israel

**Keywords:** DC/TMD, Myofascial pain with referral, Local myalgia, Axis II

## Abstract

**Background:**

The Diagnostic Criteria for Temporomandibular Disorders (DC/TMD) categorized TMD muscle disorders into 3 subgroups: local myalgia, myofascial pain with spreading and myofascial pain with referral. However, the rationale for such division into subgroups and the pathogenesis and prognosis of muscle-related TMD are still poorly understood**.** The aim of this study was to explore the differences between local myalgia and myofascial pain with referral by means of a biopsychosocial model based on the DC/TMD.

**Methods:**

This retrospective study included all consecutive TMD patients who were diagnosed according to the DC/TMD in our institution between 2015 and 2018. The Axis I and II findings of patients diagnosed with local myalgia were compared to those of patients with myofascial pain with referral. A *p* value < 0.05 was considered statistically significant.

**Results:**

A total of 255 patients (61 men and 194 women, mean age 37.8 ± 15.34 years) were enrolled into the study, 114 in the local myalgia group and 83 in the myofascial pain with referral group. The levels of depression and nonspecific physical symptoms, headache attributed to TMD (HAattrTMD), and characteristic pain intensity (CPI) were significantly higher in the latter group. The significant differences for depression and nonspecific physical symptoms persisted after excluding patients diagnosed with HAattrTMD, however, the levels of significance were lower (*p* = 0.006 compared to *p* = 0.033 for depression total score, and *p* = 0.001 compared to *p* = 0.046 for nonspecific physical symptoms total score). CPI levels, extent of disability, and pain duration were similar for both groups when excluding for HAattrTMD.

**Conclusion:**

The current study findings highlight the importance of differentiating between subgroups of myalgia according to the DC/TMD. The diagnosis of myofascial pain with referral may point to a significant Axis II component.

## Background

Temporomandibular disorders (TMD) are defined as "a group of musculoskeletal and neuromuscular conditions that involve the temporomandibular joints (TMJs), the masticatory muscles, all associated structures of mastication, and associated tissues" [1]. Diagnostic criteria based on a biopsychosocial model [the Research Diagnostic Criteria/TMD (RDC/TMD) and the newer version, the DC/TMD] enabled exploration of the association of both physical aspects (Axis I) and psychological factors (Axis II) in order to understand the development of cases of persistent TMD that are refractory to conventional treatment.

Muscle-related disorders represent the largest subgroup among the various TMD diagnoses [2]. The RDC/TMD has included only one diagnosis of muscle-related disorders for research purposes, that of "myofascial pain with/without limited opening". Following the RDC/TMD validation project [3], the newer DC/TMD [4] revised portions of both the Axis I and Axis II components. One of the changes was the categorization of TMD muscle disorders into the 3 subgroups of local myalgia, myofascial pain with spreading, and myofascial pain with referral. Among them, local myalgia and myofascial pain with referral received acceptable levels of sensitivity and specificity for a definitive diagnosis (a sensitivity of 90% and a specificity of 99% for local myalgia, and a sensitivity of 86% and a specificity of 98% for myofascial pain with referral). However, neither diagnosis has been assessed for criterion validity [5].

The 2 diagnoses are closely related to jaw function and differ only by the ability to create local versus referred pain beyond the boundary of the muscle being palpated. As such, the basis for differentiating between them is purely clinical. It should be noted that although diagnostic criteria for all subgroups of muscle-related pain were established in the DC/TMD, the pathogenesis and prognosis of muscle-related TMD is still poorly understood. Several central and peripheral theories have attempted to explain the pathophysiology underlying myofascial pain with referral [6–9]. The pathophysiology underlying chronic local myalgia is unclear as well [10–13]. This uncertainty of the pathophysiology of muscle-related TMD is reflected in the first edition of the International Classification of Orofacial Pain (ICOP) which was recently released [14]. The authors of the ICOP proposed adherence to the single terminology of "myofascial" in order to reflect the yet undetermined etiology.

However, so far only few studies have examined the differences between local myalgia and myofascial pain with referral by applying the DC/TMD [15–17]. There are several studies on the differences in treatment response between the 2 diagnoses [18, 19]. Other studies which used the DC/TMD did not differentiate between the different subgroups of muscle-related disorders [20, 21].

The current study was performed following the recommendation of the co-lead authors [22, 23] of the DC/TMD [4] to determine whether there is any difference between the subgroups of myalgia according to the DC/TMD in terms of mechanisms and clinical implications. Therefore, the purpose of this study was to further explore the differences between local myalgia and myofascial pain with referral, as to Axis I and II of the DC/TMD, according to the biopsychosocial model.

## Methods

The study was approved by the Tel Aviv University Institutional Ethical Committee prior to data collection (#14134_20180327). Informed consent was waived since the data were retrieved retrospectively. However, each patient who is referred to the "Orofacial and TMD Clinic" routinely signs a form in which s/he agrees that his/her data can be anonymously used for research purposes. The study was self-funded by the authors.

This retrospective study population included 558 consecutive patients who were seen for the first time in the Tel Aviv University Orofacial Pain Clinic during 2015–2018. All the patients were examined by senior staff members certified in the DC/TMD Training and Calibration Course at the Department of Orofacial Pain and Jaw Function at the Faculty of Odontology at Malmö University, Sweden. Each patient who was suspected as having TMD underwent a full DC/TMD Axis I and Axis II evaluation according to the official Hebrew version [24] of the DC/TMD [4]. The nonpainful Axis I diagnoses included intra-articular TMD (disc displacement with reduction, disc displacement with reduction with intermittent locking, disc displacement without reduction with limited opening, and disc displacement without reduction without limited opening), degenerative joint disease, and subluxation. Painful Axis I diagnoses included arthralgia, HAattrTMD, local myalgia, and myofascial pain with referral.

**Fig. 1:**
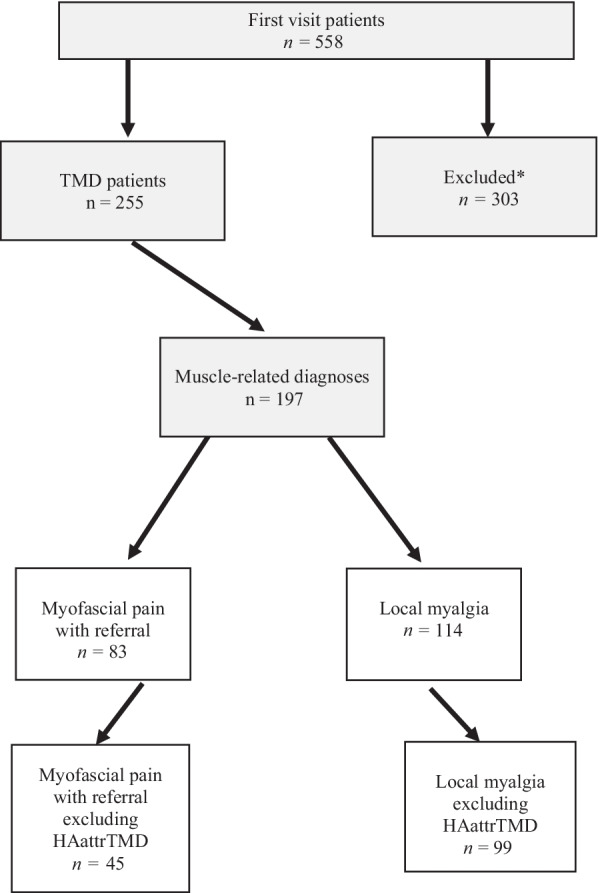
Flow chart of study groups. *Excluded from the study were 56 patients who did not complete the DC/TMD questionnaire, 67 patients who were younger than 18 years, 142 patients who received other orofacial pain diagnoses that included systemic diseases (e.g., fibromyalgia and inflammatory arthritis), and 38 patients who did not meet the DC/TMD criteria for diagnosis of TMD

The following instruments were used to evaluate Axis II: depression level [Patient Health Questionnaire (PHQ)-9], nonspecific physical symptom levels (PHQ-15 questionnaire for somatic symptoms), anxiety level [Generalized Anxiety Disorder (GAD)-7]. Characteristic pain intensity (CPI), pain persistence classification, and Graded Chronic Pain Scale (GCPS) version 2.0 were calculated for each patient according to the specifications of the DC/TMD. Excluded from the study were 56 patients who did not complete the DC/TMD questionnaire, 67 patients who were younger than 18 years, 142 patients who received other orofacial pain diagnoses that included systemic diseases (e.g., fibromyalgia and inflammatory arthritis), and 38 patients who did not meet the DC/TMD criteria for diagnosis of TMD, leaving a total 255 TMD patients. The final study population was comprised of 197 patients who received Axis I muscle-related diagnosis, 114 of whom were diagnosed as having local myalgia and 83 patients diagnosed as having myofascial pain with referral (Fig. 1).

### Statistical analysis

Continuous variables were evaluated for normal distribution by means of a histogram and Q-Q plots. Since the continuous variables did not distribute normally, they were reported as the median and interquartile range (IQR) and standard deviation (SD) and analyzed by non- parametric tests. Categorical variables were described as frequency and percentage. The Pearson chi-squared test and Fisher's Exact test were used to test the associations between categorical variables. The Mann–Whitney test was used to assess differences in continuous variables between categories. All tests were 2-tailed. IBM SPSS Statistics for Windows, Version 25.0. Armonk, NY: IBM Corp. was used for all statistical analyses. A *p* value < 0.05 was considered statistically significant.

## Results

Overall, among the 255 TMD patients, the female:male ratio was 3:1, and the mean age ± SD was 37 ± 15.3 years. The Axis I painful diagnoses were local myalgia in 114 patients (44.7%), myofascial pain with referral in 83 patients (32.5%), arthralgia in 50 patients (19.6%), and HAattrTMD in 60 patients (23.5%). The Axis II results showed that 42 patients (16.5%) reported high levels of disability (GCPS levels 3–4), 20 (7.8%) scored moderately severe-severe on the depression questionnaire (PHQ-9), 13 (5.1%) scored severe on the anxiety questionnaire (GAD-7), and 10 (3.9%) scored severe on the nonspecific physical symptoms questionnaire (PHQ-15). 70 (27.5%) of the patients reported persistent pain in the last 30 days.

In the first stage of the study, only patients with muscle-related disorders were included. They were divided into 1 group diagnosed as having local myalgia (*n* = 114) and a second group diagnosed as having myofascial pain with referral (*n* = 83). The demographic and socioeconomic characteristics of the 197-patient cohort are listed in Table 1. The only significant difference between the 2 groups was income (*p* = 0.043). Only 5 (6.3%) of the myofascial pain with referral group reported very low-low income, compared to 18 (16.7%) of the local myalgia group.

**Table 1:** Demographic and socioeconomic data of the 2 study groups (local myalgia vs. myofascial pain with referral)

	Local myalgia	Myofascial pain with referral	*p*
Sex			0.165
Male	22 (19.3%)	23 (27.7%)	
Female	92 (80.7%)	60 (72.3%)	
Age (years)			0.813
(Mean ± SD)	39.00 ± 16.73	38.13 ± 14.45	
Median (IQR)	32.50 (25.75–51.00)	34.00 (28.00–52.00)	
Education			0.984
Elementary/high school	38 (33.6%)	27 (32.9%)	
Some college/college graduate	50 (44.2%)	36 (43.9%)	
Professional or post-graduate level	25 (22.1%)	19 (23.2%)	
Income			**0.043**
Very low, low	18 (16.7%)	5 (6.3%)	
Average	67 (62.0%)	49 (61.3%)	
High, very high	23 (21.3%)	26 (32.5%)	
Marital status			0.415
Never married	46 (40.7%)	33 (39.8%)	
Married/living as married	58 (51.3%)	47 (56.6%)	
Divorced/separated	5 (4.4%)	3 (3.6%)	
Widowed	4 (3.5%)	0 (0%)	

*Significant values are shown in bold

Table 2 presents the pain characteristics of the 2 study groups. There were no differences between them in pain duration (*p* = 0.217), headache duration (*p* = 0.295), and pain persistence score (*p* = 0.363). However, the myofascial pain with referral group reported significantly more headaches during the preceding 30 days (*p* = 0.002) and higher pain intensity (*p* = 0.033).

**Table 2:** Pain characteristics of the 2 study groups (local myalgia vs. myofascial pain with referral)

	Local myalgia	Myofascial pain with referral	*p*
Temporal characteristics: In the last 30 days, which of the following best describes any pain in your jaw, temple, in the ear, or in front of the ear on either side?			
No pain	5 (4.5%)	2 (2.4%)	0.772
Pain comes and goes	72 (64.9%)	52 (63.4%)	
Pain is always present	34 (30.6%)	28 (34.1%)	
Pain duration: How many months ago did your pain in the jaw, temple, in the ear, or in front of the ear first begin?			
(Mean ± SD)	50.08 ± 73.01	61.03 ± 93.93	0.217
Median (IQR)	18.00 (6.00–60.00)	24.00 (11.50–72.00)	
Report of headache: In the last 30 days, have you had any headaches that included the temple areas of your head?			
No	53 (46.9%)	21 (25.6%)	**0.002**
Yes	60 (53.1%)	61 (74.4%)	
Headache duration: How many years or months ago did your temple headache first begin?			
(Mean ± SD)	62.73 ± 77.58	52.05 ± 98.93	0.295
Median (IQR)	27.00 (4.25–96.00)	24.00 (3.00–60.00)	
Characteristic Pain Intensity (CPI) (1–100)			
Mean ± SD)	54.27 ± 23.03	60.9 ± 21.74)	**0.033**
Median (IQR**)**	53.33 (40.00–70.00)	66.67 (43.33–80.00)	
Pain persistence: On how many days in the last 6 months have you had facial pain?			
(Mean ± SD)	80.16 ± 76.44	84.51 ± 69.89	0.499
Median (IQR)	30.00 (13.25–180.00)	60.00 (20.00–180.00)	
Pain Persistence Score:			
Low (≤ 89 days)	58 (60.4%)	39 (53.4%)	0.363
High (≥ 90 days)	38 (39.6%)	34 (46.6%)	

*Significant values are shown in bold

Table 3 presents the distribution of Axis I diagnoses in the 2 study groups. The only significant difference between them was a higher prevalence of the diagnosis of HAattrTMD in the myofascial pain with referral group (*p* < 0.001).

**Table 3:** Axis I diagnoses of the 2 study groups (local myalgia vs. myofascial pain with referral)

	Local myalgia	Myofascial pain with referral	*p*
Headache attributed to TMD	15 (13.2%)	38 (45.8%)	**< 0.001**
Disc displacement	43 (37.7%)	28 (33.7%)	0.565
DJD	18 (15.8%)	11 (13.3%)	0.620
Arthralgia	25 (21.9%)	18 (21.7%)	0.967
Subluxation	16 (14.0%)	9 (10.8%)	0.506

*Significant values are shown in bold

Table 4 lists the Axis II findings of both groups. Significant differences were found in the total scores for PHQ-9 (depression) (*p* = 0.006) and PHQ-15 (nonspecific physical symptoms; *p* = 0.001), which were significantly higher in the myofascial pain with referral group. Significant differences were found in interference score (*p* < 0.001). The 2 groups did not differ in their disability scores [21 (18.8%) patients in the local myalgia group had high scores in the category of disability (GCPS 3 and 4), compared to 17 (20.5%) patients in the myofascial pain with referral group.] (*p* = 0.763).

**Table 4:** Axis II diagnoses of the 2 study groups (local myalgia vs. myofascial pain with referral)

		Local myalgia	Myofascial pain with referral	*p*
GCPS version 2.0				
Low disability (GCPS 0–2)		91 (81.3%)	66 (79.5%)	0.763
High disability (GCPS 3–4)		21 (18.8%)	17 (20.5%)	
Interference score				
(Mean ± SD)		24.62 ± 30.25	30.52 ± 29.03	**< 0.001**
Median (IQR)		10.00 (0.00–36.66)	20.00 (6.66–50.00)	
PHQ- 9 (Depression)				
Normal	≤ 4	64 (56.1%)	35 (42.2%)	0.258
Mild	5–9	32 (28.1%)	31 (37.3%)	
Moderate	10–14	9 (7.9%)	10 (12.0%)	
Moderately severe-severe	15 +	9 (7.9%)	7 (8.4%)	
PHQ—9 total score				
(Mean ± SD)		5.08 ± 5.40	6.61 ± 5.15	**0.006**
Median (IQR)		2.00 (0.00–4.00)	6.00 (3.00–8.00)	
GAD-7 (Generalized anxiety)				
Normal	≤ 4	78 (68.4%)	47 (56.6%)	0.272
Mild	5–9	21 (18.4%)	25 (30.1%)	
Moderate	10–14	9 (7.9%)	7 (8.4%)	
Severe	15 +	6 (5.3%)	4 (4.8%)	
GAD- 7 total score				
(Mean ± SD)		3.91 ± 4.65	4.66 ± 4.71	0.113
Median (IQR)		2.00 (0.00–6.00)	4.00 (1.00–7.00)	
PHQ- 15 (Nonspecific physical symptoms)				
Normal	≤ 4	63 (55.3%)	30 (36.1%)	0.064
Mild	5–9	34 (29.8%)	37 (44.6%)	
Moderate	10–14	12 (10.5%)	12 (14.5%)	
Severe	15 +	5 (4.4%)	4 (4.8%)	
PHQ- 15 total score				
(Mean ± SD)		4.73 ± 4.38	6.47 ± 4.24	**0.001**
Median (IQR)		4.00 (1.00–7.00)	6.00 (4.00–9.00)	

*Significant values are shown in bold

In the second stage of the study, patients diagnosed with HAattrTMD were removed from both groups, and patients with myofascial pain with referral (*n* = 45) were compared with patients with local myalgia (*n* = 99). Tables 5, 6 and 7 list the demographic, socioeconomic, pain characteristics, and Axis I findings in both groups. There were no differences between them for those parameters, including pain duration, pain levels, pain persistence, and all Axis I diagnoses.

**Table 5:** Demographic and socioeconomic data of the 2 study groups (local myalgia vs. myofascial pain with referral excluding headache attributed to TMD)

	Local myalgia (Without HAattrTMD)	Myofascial pain with referral (Without HAattrTMD)	*p*
Sex			0.115
Male	19 (19.2%)	14 (31.1%)	
Female	80 (80.8%)	31 (68.9%)	
Age (years)			0.947
Mean ± SD	40.11 ± 17.27	39.36 ± 16.07	
Median (IQR)	36.00 (26.00–52.00)	34.00 (27.00–52.00)	
Education			0.482
Elementary/high school	31 (31.6%)	18 (40.0%)	
Some college/college graduate	45 (45.9%)	16 (35.6%)	
Professional or post-graduate level	22 (22.4%)	11 (24.4%)	
Income			0.524
Very low, low	15 (16.1%)	4 (9.1%)	
Average	60 (64.5%)	30 (68.2%)	
High, very high	18 (19.4%)	10 (22.7%)	
Marital status			0.609
Never married	37 (37.8%)	18 (49.0%)	
Married/living as married	52 (53.1%)	26 (57.8%)	
Divorced/separated	5 (5.1%)	1 (2.2%)	
Widowed	4 (4.1%)	0 (0%)

**Table 6:** Pain characteristics of the 2 study groups (local myalgia vs. myofascial pain with referral excluding headache attributed to TMD)

	Local myalgia (Without HAattrTMD)	Myofascial pain with referral (Without HAattrTMD)	*p*
Temporal characteristics: In the last 30 days, which of the following best describes any pain in your jaw, temple, or ear?			
No pain	4 (4.2%)	2 (4.5%)	0.892
Pain comes and goes	66 (68.8%)	32 (72.7%)	
Pain is always present	26 (27.1%)	10 (22.7%)	
Pain duration: How many months ago did your pain in the jaw, temple, in the ear, or in front of the ear first begin?			
Mean ± SD	45.77 ± 70.11	68.37 ± 112.98	0.204
Median (IQR)	17.00 (6.0–48.00)	24 (11.00–72.00)	
Report of headache: In the last 30 days, have you had any headaches that included the temple areas of your head?			
No	53 (54.1%)	20 (45.5%)	0.342
Yes	45 (45.9%)	24 (54.5%)	
Headache duration: How many years or months ago did your temple headache first begin?			
(Mean ± SD)	56.95 ± 75.12	52.65 ± 137.22	0.259
Median (IQR)	18.00 (3.00–96.00)	12 (2.00–36.00)	
Characteristic Pain Intensity (CPI) (1–100)			
Mean ± SD)	52.75 ± 23.10	55.85 ± 23.34	0.459
Median (IQR)	50.00 (39.16–66.66)	60.00 (35.00–76.66)	
Pain persistence: On how many days in the last 6 months have you had facial pain?			
(Mean ± SD)	77.68 ± 75.20	73.35± 66.32	0.499
Median (IQR)	30.00 (13.50 -180.00)	50.00 (20.00–120.00)	
Pain Persistence Score:			
Low (≤ 89 days)	50 (61.7%)	25 (62.5%)	0.934
High (≥ 90 days)	31 (38.3%)	15 (37.5%)

**Table 7:** Axis I diagnoses of the 2 study groups (local myalgia vs. myofascial pain with referral excluding headache attributed to TMD)

	Local myalgia (Without HAattrTMD)	Myofascial pain with referral (Without HAattrTMD)	*p*
Disc displacement	36 (36.4%)	12 (26.7%)	0.253
DJD	18 (18.2%)	6 (13.3%)	0.469
Arthralgia	19 (19.2%)	10 (22.2%)	0.674
Subluxation	13 (13.1%)	5 (11.1%)	0.734

Table 8 lists Axis II diagnoses in the 2 groups. After removing the patients diagnosed with HAattrTMD, total depression scores and total nonspecific physical symptoms scores remained significantly higher in the myofascial pain with referral group, although the level of significance was lower than the findings obtained for the first stage (*p* = 0.006 compared to *p* = 0.033 for the total depression score, and *p* = 0.001 compared to *p* = 0.046 for the total nonspecific physical symptoms score).

**Table 8:** Axis II diagnoses of the 2 study groups (local myalgia vs. myofascial pain with referral excluding headache attributed to TMD)

		Local myalgia (Without HAattrTMD)	Myofascial pain with referral (Without HAattrTMD)	*p*
GCPS version 2.0				
Low disability (GCPS 0–2)		81 (83.5%)	38 (84.4%)	0.888
High disability (GCPS 3–4)		16 (16.5%)	7 (15.6%)	
Interference score				
(Mean ± SD)		22.50 ± 29.03	25.33 ± 27.66	0.221
Median (IQR)		10.00 (0.00–33.33)	16.66 (3.33–40.00)	
PHQ- 9 (Depression)				
Normal	≤ 4	61 (61.6%)	21 (46.7%)	0.376
Mild	5–9	24 (24.2%)	16 (35.6%)	
Moderate	10–14	7 (7.1%)	4 (8.9%)	
Moderately severe-severe	15 +	7 (7.1%)	4 (8.9%)	
PHQ—9 total score				
(Mean ± SD)		4.71 ± 5.34	6.38 ± 5.61	**0.033**
Median (IQR)		3.00 (1.00–7.00)	6.00 (2.00–8.00)	
GAD-7 (Generalized anxiety)				
Normal	≤ 4	69 (69.7%)	26 (57.8%)	0.501
Mild	5–9	17 (17.2%)	12 (26.7%)	
Moderate	10–14	7 (7.1%)	4 (8.9%)	
Severe	15 +	6 (6.1%)	3 (6.7%)	
GAD- 7 total score				
(Mean ± SD)		3.85 ± 4.78	4.73 ± 5.23	0.261
Median (IQR)		2.00 (0.00–5.00)	4.00 (0.00–7.00)	
PHQ- 15 (Nonspecific physical symptoms)				
Normal	≤ 4	58 (58.6%)	20 (44.4%)	0.385
Mild	5–9	28 (28.3%)	18 (40.0%)	
Moderate	10–14	10 (10.1%)	5 (11.1%)	
Severe	15 +	3 (3.0%)	2 (4.4%)	
PHQ- 15 total score				
(Mean ± SD)		4.34 ± 4.20	5.71 ± 4.30	**0.046**
Median (IQR)		3.00 (1.00–7.00)	6.00 (1.50–8.00)	

*Significant values are shown in bold

## Discussion

The purpose of this study was to explore the differences between local myalgia and myofascial pain with referral as measured by the biopsychosocial model according to the DC/TMD. The results suggest that TMD patients diagnosed with myofascial pain with referral may have a significant Axis II component. These results support the findings of a recent study by Barjandi et al. [17] that showed that TMD patients diagnosed with local myalgia scored significantly lower in the domains of depression, anxiety, somatic symptoms, pain catastrophizing, perceived stress, sick days, and insomnia compared to TMD patients diagnosed with myofascial pain with referral. Barjandi et al. [17] suggest therefore that a diagnosis of myofascial pain with referral could be indicative of a more severe condition compared to a diagnosis of local myalgia and may even point to a potential transition to fibromyalgia. It is the authors' belief that the findings of the current study demonstrate that using myalgia as the sole muscle-related diagnosis according to the DC/TMD without examining the subgroup diagnoses separately (i.e., local myalgia, and myofascial pain with referral) may be incomplete for clinical and research application.

Indeed, numerous studies have suggested that myofascial pain with referral results in the induction of central sensitization after ongoing peripheral nociceptive stimuli [25–28], implying the possibility of transference to a chronic pain condition. There are also many studies that support an association between myofascial pain with referral and Axis II parameters [29–34]. Studies that have consistently shown that chronic TMD patients scored higher levels of Axis II on psychological questionnaires [35–38]. Moreover, prospective studies have shown that psychological variables predicted an increased risk for developing TMD [39, 40], and that the use of Axis II evaluation through the RDC/TMD for psychosocial assessment can aid in clinical decision making for the management of TMD [41].

Importantly, the current study demonstrated higher levels of Axis II irrespective of pain duration and pain intensity. This highlights that aside from pain duration and intensity other components contribute to persistence of TMD, such as genetic factors, environmental contributions, life stressors, autonomic function, and impaired pain regulation [42–44].

In the current study, the more widespread the pain (including HAattrTMD diagnosis) the more apparent were the differences in pain level, and the greater was the increase in the level of significance of the Axis II findings, including interference score which was higher in the myofascial pain with referral group which included patients with diagnosis of HAattrTMD. While no findings in disability levels and pain duration were found in the current study, it was previously shown that the diagnosis of HAattrTMD was associated with myofascial pain with referral, significantly higher Axis II results, greater disability, and higher pain levels, but no differences in pain duration compared to painful TMD patients without diagnosis of HAattrTMD [15]. These findings are consistent with those of others that associated widespread pain with significant Axis II components [41, 45, 46] and disability [47–49].Early identification of patients who are at a higher risk for developing chronic TMD may therefore aid in developing an effective tailored early intervention to reduce potential transference to a chronic condition.

The current study findings of significantly higher Axis II levels in the myofascial pain with referral group also raise the possibility of identifying potential future chronic TMD patients as early as the first visit with the sole use of Axis I findings of myofascial with referral according to the DC/TMD. This may be of significant clinical value, especially in situations where Axis II information is not available, and only Axis I information is used for diagnosis according to the DC/TMD [18, 19, 50].

The current study could not answer the question of whether local myalgia and myofascial pain with referral represent different entities. As observed by Michelotti et al. [23], it is possible that both entities are presentations of a single disorder and therefore represent a continuum from mild and remittent local pain to more regional and continuous severe pain. On the other hand, it is possible that they are entirely separate disorders, a notion that is supported by others who believe that muscle-related TMD as a group encompasses a number of conditions [2]. It should be emphasized that the criterion validity of these muscular subgroups has not yet been established [5]. Future studies are needed in order to further examine the above hypotheses, especially those pertaining to findings that raise the possibility of transformation to a chronic pain disease.

## Conclusions

The current study highlights the importance of differentiating between subgroups of myalgia according to the DC/TMD, specifically local myalgia and myofascial pain with referral, for both clinical and research purposes. The findings suggest that a diagnosis of myofascial pain with referral may be indicative of a significant Axis II component which may require a tailored treatment approach in order to try to prevent transference to a chronic pain condition. Finally, a higher score of an Axis II component may be expected in association with more widespread pain, including HAattrTMD.

## Data Availability

The datasets used and/or analyzed during the current study is available from the corresponding author on reasonable request.

## Abbreviations

DC/TMD: Diagnostic Criteria for Temporomandibular Disorders
HAattrTMD: Headache attributed to TMD
CPI: Characteristic pain intensity
TMD: Temporomandibular disorders
TMJs: Temporomandibular joints
RDC/TMD: Research Diagnostic Criteria/TMD
ICOP: International Classification of Orofacial Pain
PHQ: Patient Health Questionnaire
GAD: Generalized Anxiety Disorder
GCPS: Graded Chronic Pain Scale
